# Supplementary material for: Economic Evaluations of Magnetic Resonance Image-Guided Radiotherapy (MRIgRT): A Systematic Review
Source: Int J Environ Res Public Health. 2022 Aug 30;19(17):10800. doi: 10.3390/ijerph191710800 (PMC9517760; doi:10.3390/ijerph191710800)
Supplement: Supplementary file 1 [file ijerph-19-10800-s001.zip › ijerph-1876906-supplementary.pdf]

Supplementary Material

Search on 7 March 2022, **Ovid MEDLINE(R) and Epub Ahead of Print, In-Process, In-Data-Review & Other Non-Indexed Citations, Daily and Versions 1946 to March 04, 2022**

# ▲	Searches	Results
1	((Multiparametric Magnetic Resonance Imaging/ or Magnetic Resonance Imaging/ or Magnetic Resonance Imaging, Interventional/ or (Magnetic Resonance or MR or NMR or MRI).af.) and (guidance or guided or IG or IGRT or simulat*).af.) or (MR-guided or MRI-guided).af.	69659
2	(RADIOTHERAPY or RADIATION or RT or ART or personali*).af.	1454386
3	(MRgART or MRgRT or MRIgRT).af.	243
4	(1 and 2) or 3	8106
5	"Costs and Cost Analysis"/	50439
6	Cost-Benefit Analysis/ or (Cost-Benefit or Cost-Effectiveness or Cost-Utility or Economic Evaluation* or cost or costs or costing).af.	738983
7	Health Care Costs/	42950
8	Direct Service Costs/	1217
9	Employer Health Costs/	1097
10	Hospital Costs/	11781
11	Economic Models/ or Economic Model*.af.	13648
12	5 or 6 or 7 or 8 or 9 or 10 or 11	742761
13	4 and 12	293

Search on **Scopus** (07/03/2022)

(
(
(
(
(
TITLE-ABS-KEY ("Magnetic Resonance")
OR TITLE-ABS-KEY (mr)
OR TITLE-ABS-KEY (nmr)
OR TITLE-ABS-KEY (mri)

)
AND (
TITLE-ABS-KEY (guidance)
OR TITLE-ABS-KEY (guided)
OR TITLE-ABS-KEY (ig)
OR TITLE-ABS-KEY (igrt)
OR TITLE-ABS-KEY (simulat*))
)
OR TITLE-ABS-KEY (mr-guided)
OR TITLE-ABS-KEY (mri-guided)
)
AND (
TITLE-ABS-KEY (radiotherapy)
OR TITLE-ABS-KEY (radiation)
OR TITLE-ABS-KEY (rt)
OR TITLE-ABS-KEY (art)
OR TITLE-ABS-KEY (personali*)
)
)
OR (
TITLE-ABS-KEY (mrgart)
OR TITLE-ABS-KEY (mrgrt)
OR TITLE-ABS-KEY (mrigrt)
)
)
AND (
TITLE-ABS-KEY (cost)
OR TITLE-ABS-KEY (costs)
OR TITLE-ABS-KEY (costing)
OR TITLE-ABS-KEY (cost-benefit)
OR TITLE-ABS-KEY (cost-effectiveness)
OR TITLE-ABS-KEY (cost-utility)
OR TITLE-ABS-KEY (economic AND evaluation*)
OR TITLE-ABS-KEY (economic AND model*)
)

Papers found: 622

Search on **INAHTA** (https://database.inahta.org/) on 7 March 2022

((((Multiparametric Magnetic Resonance Imaging)[mh] or (Magnetic Resonance Imaging)[mh] or (Magnetic Resonance Imaging, Interventional)[mh] or Magnetic Resonance or MR or NMR or MRI) and (guidance or guided or IG or IGRT or simulat*)) or MR-guided or MRI-guided) AND (RADIOTHERAPY or RADIATION or RT or ART or personali*)) OR (MRgART or MRgRT or MRIgRT)

Papers found: 51
